# Calibrating the Discrete Boundary Conditions of a Dynamic Simulation: A Combinatorial Approximate Bayesian Computation Sequential Monte Carlo (ABC-SMC) Approach

**DOI:** 10.3390/s24154883

**Published:** 2024-07-27

**Authors:** Jah Shamas, Tim Rogers, Anton Krynkin, Jevgenija Prisutova, Paul Gardner, Kirill V. Horoshenkov, Samuel R. Shelley, Paul Dickenson

**Affiliations:** 1 Department of Mechanical Engineering, University of Sheffield, Mappin Street, Sheffield S1 3JD, UK; jshamas1@sheffield.ac.uk (J.S.); tim.rogers@sheffield.ac.uk (T.R.); j.prisutova@sheffield.ac.uk (J.P.); p.gardner@sheffield.ac.uk (P.G.); k.horoshenkov@sheffield.ac.uk (K.V.H.); 2 nuron Ltd., Riverbank House, 2 Swan Lane, London EC4R 3TT, UK; samuel.shelley@nuron.tech (S.R.S.); paul.dickenson@nuron.tech (P.D.)

**Keywords:** Bayesian inference, Monte Carlo simulation, structural vibration, uncertainty quantification

## Abstract

This paper presents a novel adaptation of the conventional approximate Bayesian computation sequential Monte Carlo (ABC-SMC) sampling algorithm for parameter estimation in the presence of uncertainties, coined *combinatorial* ABC-SMC. Inference of this type is used in situations where there does not exist a closed form of the associated likelihood function, which is replaced by a simulating model capable of producing artificial data. In the literature, conventional ABC-SMC is utilised to perform inference on continuous parameters. The novel scheme presented here has been developed to perform inference on parameters that are high-dimensional binary, rather than continuous. By altering the form of the proposal distribution from which to sample candidates in subsequent iterations (referred to as waves), high-dimensional binary variables may be targeted and inferred by the scheme. The efficacy of the proposed scheme is demonstrated through application to vibration data obtained in a structural dynamics experiment on a fibre-optic sensor simulated as a finite plate with uncertain boundary conditions at its edges. Results indicate that the method provides sound inference on the plate boundary conditions, which is validated through subsequent application of the method to multiple vibration datasets. Comparisons between appropriate forms of the metric function used in the scheme are also developed to highlight the effect of this element in the schemes convergence.

## 1. Introduction

The calibration of complex computer models remains a challenging problem within engineering contexts. This issue is exacerbated by two factors: high dimensionality, and lack of differentiability in the parameter space of interest. A methodology for targeting the combination of these two problematic scenarios is the key contribution of this work. Additionally, in almost all scenarios, aligning the outputs of a computer model (simulator) with measured data is complicated by the presence of uncertainties throughout the process, some of which may be reducible (e.g., model parameters such as material properties) or irreducible (e.g., pollution of measured data by noise). The understanding and quantification of this uncertainty is often a key step in the calibration process, and one where problems of high dimensionality and lack of differentiability are exacerbated.

In this work, the authors adopt a Bayesian standpoint on the calibration problem, where the user seeks to find a posterior distribution over the parameters of interest given the combination of evidence from observed data and expert judgement (expressed as prior belief). This Bayesian viewpoint on uncertainty is by no means the only approach for the quantification of uncertainty; however, it has previously been shown to be effective in problems the readers will be familiar with. In the context of structural dynamics, there exist several known methods appealing to Bayes’ theorem to infer structural parameters [1,2,3]. These have proved to be popular avenues for engineers to take when attempting to perform inference on parameters of dynamical systems when working with measured data. It is also widely known that Bayesian methodologies can be adopted in the context of structural model updating. For example, the works of Ching and Chen [4], Cheung and Beck [3], Lye et al. [5] all show effective methodologies for the recovery of Bayesian posteriors. Just as the successes of the Bayesian approach are well known, so are some of the shortcomings, particularly the difficulty of inference in high dimensions; see [6].

In the examples above, the parameters of interest, such as natural frequency, exist as a set of continuous variables. Less attention has been given to problems where the variables take discrete values or where the parameter vector θ contains a mixture of continuous and discrete values. This problem of inference over discrete or mixed spaces has received comparatively less attention in general—notable exceptions being in mixture modelling or switching cases, e.g., [7,8,9]. However, in the context of engineering modelling, cases such as these may be encountered in a number of scenarios as seen in [10], where the authors used Bayesian optimisation to inform structural design of a welded beam, encoding welding types as a binary variable, whilst geometric considerations were encoded as continuous variables. The contribution of this paper is to propose a methodology for (approximate) Bayesian inference over computer models (simulators), where the parameters of interest are discrete (or mixed) and the parameter space is high dimensional. The approach is one of a sequential approximate Bayesian computation scheme with proposal kernels developed in a similar manner to that seen in Population Monte Carlo [11]. This allows for inference to be made over a thirty-six dimensional binary parameter space in the case study modelling a fibre-optic sensor housing articulation response in an efficient manner.

Commonly, Bayesian inference will be intractable, owing to lack of access to the marginal likelihood which normalises the posterior; hence, most modern Bayesian approaches will resort to an approximation of the posterior either via sampling methods such as Markov Chain Monte Carlo (MCMC), importance sampling, or sequential Monte Carlo, or via parametric approximation as in variational inference [12]. In practice, however, even the evaluation of the likelihood function—modelling the measured outputs *Y* as a function of known inputs *X*, parameterised by θ—may prove to be impractical. A closed form of a function $f_{\theta }:X\to Y$ may be available, but the form of the noise model may not be known, or $f_{\theta }$ may be known to only be an approximation of the true physical mechanisms at play due to the lack of sufficient knowledge of the system or the computational expense [13]. A collection of approaches coined *likelihood-free* methods have been produced that allow for computation of the posterior in situations where the corresponding likelihood function is unavailable. One such likelihood-free method that has proved popular within the last two decades is approximate Bayesian computation (ABC) [14]. This algorithm relies on the user being able to produce simulated data $Y^{*}$ through a simulating model with inputs θ sampled from some known distribution, e.g., the prior. The simulating model mimics the unknown likelihood function by considering the closeness of the outputs of this model to the measured data. Acceptance of θ into the approximate posterior is conditional on simulated and measured data $Y^{*}$, *Y* being similar ‘enough’ under some definition of distance [13]. The application of this algorithm and its augmented versions such as ABC-MCMC (Markov Chain Monte Carlo) [15] and ABC-SMC [16] can be seen throughout many areas, including genetics [17,18,19] and applied dynamical systems. For example, in [20], the authors demonstrated the value of ABC-SMC for parameter estimation in nonlinear system identification. In [21], the authors used both ABC-SMC and ABC-MCMC to estimate the parameters in the deterministic Lotka–Volterra model [22] to demonstrate the decreased computational cost incurred with these adapted schemes compared to traditional ABC. To the best of the author’s knowledge, all documented uses of the ABC-SMC scheme have been produced to estimate continuous parameters.

Particularly in practical engineering cases, parameters encoding the design of some structure to be assessed may manifest as binary variables as highlighted above. In such cases where likelihood-free methods are the best form of attack, the procedure in question must be assessed in order to best ascertain how the sampling of these parameters can be altered to achieve efficient model calibration from the prior to the posterior distribution through the use of Bayes’ theorem. The presented work provides an adapted version of the well-known ABC-SMC scheme in the case where the parameters to be inferred are high-dimensional sets of binary variables rather than continuous variables. In this case, it is the resampling procedure contained in the sequential component of the algorithm that has been modified to accommodate this parameter change, allowing other components of the algorithm to operate as intended. The adapted scheme is not just applicable to the application shown here but in any scenario when an altered approach of well-known likelihood-free methods is needed to suit the nature of the problem to be addressed.

In Section 2 and Section 3, the ABC, ABC-SMC, and adapted combinatorial ABC-SMC algorithms are presented. Section 4 presents a niche form of uncertainty that arose in a structural dynamics experiment considered by the authors, as well as details of numerical modelling of the experiment. Section 5 presents results from utilising the developed methodology to quantify the experimental uncertainty, followed by the interpretation of these results and notes on potential future work in Section 6.

## 2. Approximate Bayesian Computation

The already discussed Bayesian strategy for learning about unknown parameters in engineering models has been suggested as a valuable approach, owing to its formal incorporation of expert knowledge (through the prior distribution) and added insight from recovery of the full distribution over θ. It stated that it is generally possible—either exactly or numerically—to recover the posterior distribution $p\left(\;\theta \;|\;Y\;;X\;\right)$, which is the main object of interest. However, it is simple to imagine a case where the modeller has insufficient knowledge to construct the likelihood of the model $p\left(\;Y\;|\;\theta \;;X\;\right)$. This situation may arise from (broadly speaking) two areas of ignorance. First, it may not be possible to write down the correct form of $f_{\theta }$, for example, using a computer model such as a finite element simulation of a physical object that will result in some mismatch between the output of the model and the ‘true’ physical behaviour of the system. This mismatch will be termed *model discrepancy*, which will lead to an incorrect likelihood distribution and result in a biased estimation of the posterior. The second source of ignorance is regarding the form of the uncertainty on the measurements *Y*. It is commonly difficult to express as a probability distribution the uncertainty associated with the measurements, and often, assumptions will be made about the form of this uncertainty for convenience—most regularly, models of the form $p\left(\;Y\;|\;\theta \;;X\;\right)=N\left(f_{\theta }\;|\;\sigma \right)$, i.e., additive Gaussian (white) noise with standard deviation σ determined by the probabilistic specification of the problem, may be assumed. Incorrect assumption regarding the uncertainty/noise process will, similarly to model discrepancy, lead to a bias in the estimation of $p\left(\;\theta \;|\;Y\;;X\;\right)$.

The challenge in accurately specifying the likelihood $p\left(\;Y\;|\;\theta \;;X\;\right)$ and the known bias that misspecification can introduce has motivated the development of *Approximate Bayesian Computation* (ABC) techniques, which can return reliable results even when it is not possible to specify the likelihood exactly [23]. ABC attempts to retain the benefits of a *fully Bayesian* approach, i.e., recovering a posterior distribution which incorporates prior knowledge, by using an approximate likelihood where the computation of $p\left(\;Y\;|\;\theta \;;X\;\right)$ is replaced by some other function which describes how well the current set of θ allows the data to be modelled given $f_{\theta }$. The function which replaces the likelihood should measure in some way the distance $D(Y,Y^{*})$ between the data generated by the model for some choice of θ, $Y^{*}$, and the measured data *Y*. There are some restrictions on the choice of this distance function; however, in general, it is far easier to specify $D(Y,Y^{*})$ than it would be to specify a correct form for $p\left(\;Y\;|\;\theta \;;X\;\right)$.

The aim of ABC is then to gather a number of samples $n_{t}$ from an approximate form of $p\left(\;\theta \;|\;Y\;;X\;\right)$ which contains within its probability mass the true posterior distribution. Recovering such a set of samples can be achieved by employing a *rejection sampling* approach [23]. In possession of the simulating model $f_{\theta }$, one can produce outputs $Y^{*}$ for a trial set of θ values, $\theta^{\prime }$, which are the predictions from the model $f_{\theta^{\prime }}\left(X\right)$. The quality of these predictions may be assessed by the previously defined distance function $D(Y,Y^{*})$, and then, if this is below some specified threshold α, that sample could be designated as plausible and is stored. If the distance is greater than the minimum acceptable as set by α, then $\theta^{\prime }$ is discarded (rejected), and another possible trial θ is drawn. This rejection sampling procedure is outlined in Algorithm 1 [24].
**Algorithm 1:** ABC rejection sampler.
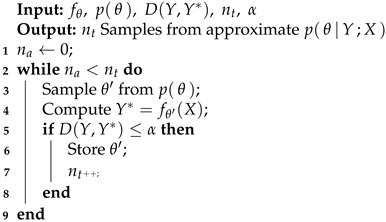


Assuming that θ∈Θ, where Θ is the space of all possible θ, one can imagine that if only a small part of Θ contains valid samples, the rejection sampling approach may be very inefficient, i.e., many guesses of θ are discarded. This issue can often be encountered in practice and is exacerbated when the computation of $Y^{*}=f_{\theta^{\prime }}\left(X\right)$ is computationally demanding. One may then seek ways in which the efficiency of Algorithm 1 could be improved through more targeted sampling of possible $\theta^{\prime }$ to be assessed. However, it is useful to retain the properties and guarantees of this most basic ABC inference algorithm.

### ABC-SMC

The desire for a more efficient ABC inference approach led to the development of ABC-SMC [16], where SMC refers to *sequential Monte Carlo*, which—as the name suggests—utilises a sequential sampling scheme to arrive at the set of valid target samples. The main challenge of effectively using the aforementioned ABC rejection sampling approach is that very few samples may be accepted at the desired threshold; most trial sets of parameters for which the simulation is run are rejected. When a potential θ is simulated and then rejected, the computational effort associated with that simulation is, in effect, wasted. Therefore, it is favourable to design algorithms which will improve the acceptance rate and therefore reduce the amount of wasted computational effort. An introduction, more generally, to SMC for Bayesian inference can be found in [25]; however, a brief introduction to ABC-SMC is given here.

ABC-SMC attempts to increase the acceptance rate of samples through generating multiple intermediate distributions which bridge the gap between the prior and the posterior, each of these may be referred to as a population. These intermediate distributions are formed by sequentially reducing the threshold below which samples are accepted such that in earlier populations of the algorithm, sets of θ whose simulated outputs lie further from the measured *Y* are still accepted. The setup for performing ABC-SMC inference is very similar to that seen for the rejection sampling approach presented in Algorithm 1; the output of the algorithm will be a set of $n_{t}$ samples of θ that produce outputs from the simulator $Y^{*}$ which lie within the threshold α in terms of their distance from the measured *Y*. Additionally, the user specifies a set of *n* thresholds given by

(1) $$\alpha =\left\{\alpha_{0},\ldots ,\alpha_{n-1},\alpha \right\},$$

which are decreasing and whose final value α is the target threshold. Notationally, for population *t*, a set of $n_{t}$ samples will be produced ${\left\{\theta_{i}^{\left(t\right)}\right\}}_{i=1}^{n_{t}}$.

On its own, this would not improve the efficiency of the overall sampling scheme; instead, the acceptance rate would simply fall with each iteration. To facilitate the improvement in acceptance rate, the SMC component of the algorithm uses the accepted samples from the previous population (after the first) to propose $\theta^{\prime }$ from a more restricted space, which, hopefully, will contain more good samples. The full procedure is described in [16], including, in particular, comments on the choice of the *backward kernel*, which have been omitted here for brevity, although a brief description of the full algorithm is given below.

As the samples are accepted, they are also assigned a weight according to some weighting function $W_{t}\left(\theta^{\prime }\right)$ which considers the likelihood of $\theta^{\prime }$ under the prior and the $D(Y,Y^{*})$ given the simulated data under $\theta^{\prime }$. In populations after the first wave, the weights are also a function of the previous weight and likelihood under the move kernel (and backward kernel). The move kernel $M_{t}\left(\left\{\theta_{i}^{\left(t\right)}\right\},\cdot \right)$ allows samples of θ to be generated for population t+1. In other words, it facilitates the sequential part of the SMC procedure, informing the samples of the next population from those currently selected. The move kernel encompasses the following steps. First, a resampling procedure selects to carry forward those samples with the highest weights (i.e., those proportional to the smallest $D(Y,Y^{*})$). After sampling, each sample weight is reset to $1/n_{t}$. The resampling step may be omitted if the diversity in the samples (commonly measured by the effective sample size, ESS) is high enough. It should be noted that it is often beneficial to reduce sample degeneracy. After resampling, the remaining samples are used to propose possible $\theta^{\prime }$ in the next population by means of a Markov kernel $K_{t}\left(\left\{\theta_{i}^{\left(t\right)}\right\},\cdot \right)$. The most common choice for this Markov kernel (and the backward kernel) is a Gaussian with zero mean and some variance which corresponds to a random walk centred on a previous sample with some perturbation, the extent of which is governed by the variance. This selection has the advantage of simplifying the calculation of $W_{t}\left(\theta^{\prime }\right)$, ensuring that the whole parameter space can be reached.

## 3. Combinatorial ABC-SMC

In the previous section, ABC and ABC-SMC schemes have been introduced. ABC-SMC aims to reduce the problem of slow convergence when the parameter space only contains a small subset of appropriate samples—this is the typical setting in an engineering model where the posterior is much tighter than the prior distribution. Typically, ABC-SMC methods have been used in situations where the parameters of interest θ are continuous variables. However, as discussed, it is often the case that parameters in engineering models will be discrete or a mixture of discrete and continuous domains; this poses a problem since the typical random walk proposals seen in ABC-SMC are no longer appropriate. Since the parameter spaces are often high dimensional, it would be beneficial to leverage the same advantages as ABC-SMC usually does, increasing acceptance rates through a series of sampling procedures with shrinking tolerances that is applicable to these binary, or mixed spaces. By considering the contributions from all elements of the variable, their individual states, and the outputs through $f_{\theta }$, it should be assessed which combination of all variable states is more likely to produce appropriate artificial data. To accommodate this challenge, a novel scheme, coined *combinatorial* ABC-SMC is presented. The root of this terminology is the fact that the algorithm is searching for the posterior over the combination of binary variables as was necessary to resolve the experimental uncertainty presented in Section 4. In the proceeding text, the parameters to be inferred shall be denoted by θ to reflect the fact they are multidimensional. It will later become apparent why this change in variable approach was needed.

Combinatorial ABC-SMC again uses a sequential sampling scheme, projecting samples through a sequence of *n* intermediate distributions with thresholds introduced in Equation (1). The proposed solution involves developing a proposal that works similarly to that seen in [11,26]. The idea is to build a proposal distribution which depends on all the accepted samples in the previous wave, and generate new samples to be assessed independently from that distribution on the current wave, for example, computing the sample mean and variance, then proposing from that Gaussian distribution as in [27]. Here, however, this is not the desired method since the variables are binary and it is not clear how a Gaussian proposal could be leveraged. Instead, the solution explored is to develop a proposal distribution that makes the following assumption; that each dimension can be treated as independent in the proposal, i.e., there is no correlation between elements in the variable. The proposal is then given by the estimated posterior probability that a given element is equal to 1 in the previous wave, which is computed in a Monte Carlo manner based on the full set of accepted samples,

(2) $$\theta_{k}^{\prime }=\left\{\begin{array}{c} 1\;\mathrm{with}\;\mathrm{probability}\;\frac{1}{n_{t}}\sum_{i=1}^{n_{t}}1_{\left\{i\right\}}\left(\theta_{k}^{\left(t\right)}\right),\\ 0\;\mathrm{otherwise} \end{array}\right.,$$

where $1_{\left\{i\right\}}\left(\theta_{k}^{\left(t\right)}\right)$ is an indicator if $\theta_{k}^{\left(t\right)}$ is equal to one in the i-th accepted sample from the previous wave. Then, $\theta_{k}^{\prime }$ is the proposed value of the $k^{\mathrm{th}}$ dimension of a potential $\theta^{\prime }$ in wave t+1, and this is proposed with the probability equal to the proportion of instances in the previous wave where that element is equal to one (i.e., $\theta_{k}^{\left(t\right)}=1$). This can be repeated for each dimension of $\theta^{\prime }$ as independence is assumed. While this may seem complicated, an intuitive explanation is that the probability that a particular element is equal to one is simply equal to the proportion which is equal to one in the previous wave. Rather than assigning weights to accepted samples, the intermediate distribution formed by the $n_{t}$ samples described by the above procedure in each wave is simply sampled from the subsequent wave. By recording which samples are more likely to produce appropriate data, which is contained in each wave’s intermediate distribution, sampling from this on the next wave ensures a more appropriate subset of the parameter space is explored.

There is a clear case where this may be a bad choice for the proposal. If, for a particular dimension of θ, there are no accepted instances where $\theta_{k}$ is not zero, then it will be impossible to ever propose that $\theta_{k}$ is not equal to zero. This will cause problems. One potential solution, if this is to occur, is to also perform tempering on the proposal distribution. Denoting the density from which a new $\theta^{\prime }$ is sampled using Equation (2) as $\tilde{q}\left(\theta^{\prime }|\Theta_{t}\right)$, then the tempered proposal density can be given by,

(3) $$q\left(\theta^{\prime }|\Theta_{t}\right)=\tilde{q}{\left(\theta^{\prime }|\Theta_{t}\right)}^{1-\phi (t)}p{\left(\theta \right)}^{\phi (t)},$$

where ϕ(t) is a monotonically reducing sequence from 1 to 0 as a function of the wave number *t*, e.g., a linearly reducing scheme. This tempering approach ensures there is some influence of the prior to maintain diversity across the waves [28]. The contribution of this paper then is to present the new proposal in (2) to be used in the move step of the ABC-SMC scheme with the intention of targeting high-dimensional sets of binary variables. An application in which this methodology has been utilised shall be introduced in the following sections.

## 4. Problem Statement

nuron Ltd., formed in 2015, is a company that aims to utilise fibre-optic sensing for leak detection and flow measurement in the UK’s wastewater pipes. Their researchers and engineers provided a fibre-optic sensor (FOS) to be used in an experiment based at the Laboratory for Verification and Validation (LVV) at the University of Sheffield. This experiment aimed to analyse the dynamic response of a fibre-optic sensor laid at the bottom of a dry half-pipe when subjected to localised mechanical excitation. For clarity, a fibre-optic sensor is a uniquely designed structure encasing a fibre-optic cable that can be laid at the bottom of the pipe interior, spanning a given section of the pipe length. The sensor was designed so any external pressure perturbations exerted on the structure are magnified by several orders of magnitude as they propagate through, ensuring the perturbations are picked up by the encased cable. In the field, it is pressure impacts due to the fluid flow above the sensor that induce signal changes in the fibre-optic cable within. These signal fluctuations change when leakages occur, allowing leaks to be detected. It should be noted, however, that for these experiments, it is the dynamic response of the sensor structure that was of interest, rather than the interior cable signal response.

**Figure 1:**
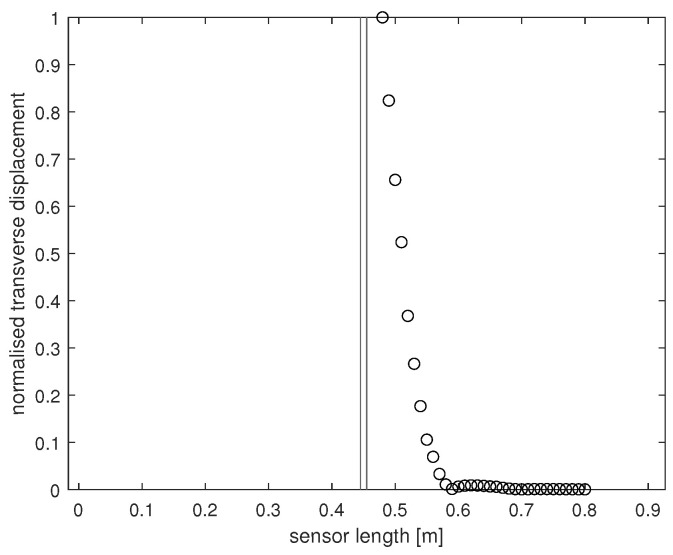
Normalised absolute displacement profile for the sensor subjected to mechanical excitation of 0.041–0.044 N operating at 5 Hz. Impact located at 45 cm along the 90 cm sensor length.

The experimental setup in the LVV facility consists of an 8 m long, 300 mm diameter clay half-pipe laid horizontally, clamped in place to minimise pipe vibration. The sensor has a length of 90 cm and consists of three separate layers. An articulation system spanning each side of the sensor was installed to ensure the sensing structure itself did not become dislodged from the pipe interior during testing. This system is divided into 18 clamp sections either side, each 5 cm in length. The coupling between the articulation system and the sensor varies between sections, and it is the application of the previously introduced methodology that resolves this uncertainty in coupling variation. The experiments at LVV involved exerting a harmonic load onto the sensor via a electrodynamic shaker at given points along the sensor length. For a given impact location, time series of the sensor transverse displacement at regular length intervals were recorded using a Laser Doppler Vibrometer (LDV). This in turn was used to build a profile of the maximum transverse displacement of the sensor for a load of given frequency, amplitude, and location. Figure 1 contains a maximum displacement profile of the sensor that was recovered through the experimental procedure described above. Note, LDV data were only collected in 1 cm intervals from 48 to 80 cm along the sensor length. This acted as a first step in understanding how nuron’s sensor would respond to simple excitation in controlled conditions.

Through the large difference between the obtained LDV data and numerical data, it became clear the extent of the coupling between the clamps and the sensing structure was unknown, implying any given edge section could move freely if the clamp at that position was not locked in place. Whatever the cause for the discrepancy between the numerical and experimental data, there was uncertainty in how to numerically model the sensor response. If each clamp is modelled as on or off, there are a potential $2^{36}$ possible clamping configurations that may be responsible for the data in Figure 1. If it took one second to numerically evaluate the maximum sensor displacement under the same experimental conditions as outlined in Figure 1 for a given clamping configuration (based on the simplified numerical model outlined in Section 4), it would take over two millennia to evaluate the displacement for all possible configurations.

The presented problem can be stated as follows: obtained experimentally are data *Y* that are dependent on some unknown parameters θ∈Θ, of which there are many. Which members of Θ may be responsible for Y? Or, in the context of the presented work, given the observed sensor response in Figure 1, what is the possible configuration of clamping that is physically responsible for these data? It is the methodology developed in the previous sections that the authors have used to resolve this boundary condition uncertainty.

### Physically Modelling the Articulation Response

**Figure 2:**
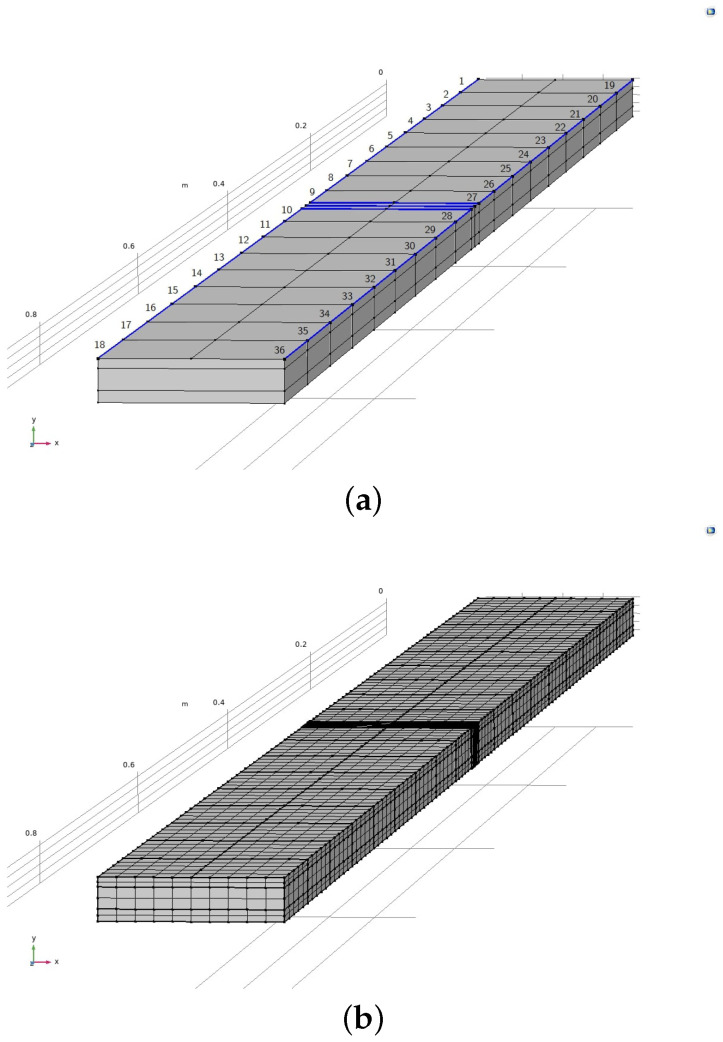
Simplified model of LVV sensor used for predicting response to mechanical excitation. Labelled clamping sections and shaker impact area are highlighted in blue in (**a**). The mesh used is displayed in (**b**).

A simplified finite element model of the LVV fibre-optic sensor was built in the finite element modelling (FEM) package COMSOL [29]. The geometry of the model is shown in Figure 2. While the actual fibre-optic sensor had a more complex geometry, through FEM simulations, it was established that simplifying the geometry has a negligible effect on sensor response; hence, it was decided to proceed with the simplified geometry that resulted in a considerable reduction in computational cost. The simplifications included not explicitly modelling the half pipe to which the sensor was attached, as well as the clamping system holding the sensor in place. The effect of these geometry elements was replicated via appropriate boundary conditions.

Note, the sections highlighted in blue that line the left and right sides of the sensors top layer in Figure 2a are where the clamps line the sensor at the pipe bottom. The finer mesh section in Figure 2b on the top of the sensor highlights where the harmonic load was exerted to replicate the data displayed in Figure 1. The top centreline of the sensor, parallel to the edges in Figure 2a, is where the maximum displacement of the sensor was plotted from, in line with the LDV measurements obtained in LVV. The presented model was used to generate simulated displacement curves $Y^{*}$ for different configurations of clamping and played a pivotal role in resolving the uncertainty of the clamping system. The following section displays the results obtained from utilising the displayed finite element model as a simulating function in the combinatorial ABC-SMC scheme.

## 5. Results and Discussion

Before displaying the results of this work, it shall be demonstrated how combinatorial ABC-SMC has been applied to resolve the physical uncertainty presented in the previous section. The approach taken was to model the clamping configuration for the sensor as a high-dimensional binary variable θ∈Θ, where $\Theta ={\left\{0,1\right\}}^{36}$. Each of the 36 elements correspond to a particular clamping section such that each element of θ is either 0 (unclamped) or 1 (clamped), resulting in the corresponding edge section being fixed or free, respectively. The authors believe that 5 cm is a good enough length for the sensor displacement to decrease (as seen in Figure 1) so that a specific clamp section may not be affected by the condition of its neighbours, and so independence between clamping sections was assumed. There is currently no known method of recovering the transverse displacement of the sensor when subjected to harmonic excitation with the complex boundary conditions that are presented here, making a likelihood function unobtainable. To circumvent this problem, simulated data $Y^{*}$ can be produced through use of the model displayed in Figure 2 that replicates the data *Y* shown in Figure 1 for the same type of excitation, with a differing configuration of boundary conditions for each simulation. Under a suitable user-defined metric *D* that not only defines the distance between the two data curves but characterises their similarity, the suitability of simulated curves and the corresponding boundary conditions can be assessed. By repeating this and projecting samples through intermediate distributions, an approximate form of the posterior distribution $p\left(\;\theta \;|\;Y\;;X\;\right)$ can be produced. This gives the probability of each boundary condition being fixed or free, given data *Y*.

Starting with a uniform probability of all boundary conditions being fixed or free, a configuration of conditions is sampled from Θ. Each of these boundary conditions are inserted into the COMSOL model, which analyses the sensor displacement in the same fashion as in the experiment. Under the user-defined metric, the experimental and simulated curves are compared; if they agree within a certain margin of error, the boundary conditions from the simulation are accepted. Otherwise, that configuration of conditions is rejected, and a different configuration of conditions is sampled. The process is repeated multiple times, sampling from the previous waves distribution, using a smaller margin of error each time, until 500 appropriate samples are accepted. During the testing of this methodology applied to the experimental data, it was found that five waves of combinatorial ABC-SMC was sufficient to achieve the target threshold α from Equation (1) and converge to realistic posterior distributions on the parameters of interest, and so the scheme was implemented over five waves for this work. Inference of the true configuration of boundary conditions would not be achievable by use of classical ABC-SMC due to how the boundary conditions manifest as random variables. The authors hope this application demonstrates how the adapted combinatorial ABC-SMC scheme can be utilised in practice to perform inference on binary rather than continuous variables in an engineering context.

Combinatorial ABC-SMC was applied twice to the data in Figure 1. In each instance, a different metric was used to characterise the similarity between normalised experimental data *Y* and normalised simulated data $Y^{*}$. It is noted that both datasets are normalised by the maximum value restricting values to the interval [0,1]. For a sampled set of boundary conditions in the first instance, the areas beneath the experimental curves *Y* in Figure 1 and $Y^{*}$, denoted as A(Y) and $A(Y^{*})$ respectively, were calculated on the interval 48–80 cm. The corresponding metric used for this run takes the form

(4) $$D\left(Y,Y^{*}\right)=\frac{|A\left(Y\right)-A\left(Y^{*}\right)|}{A(Y)}.$$

**Figure 3:**
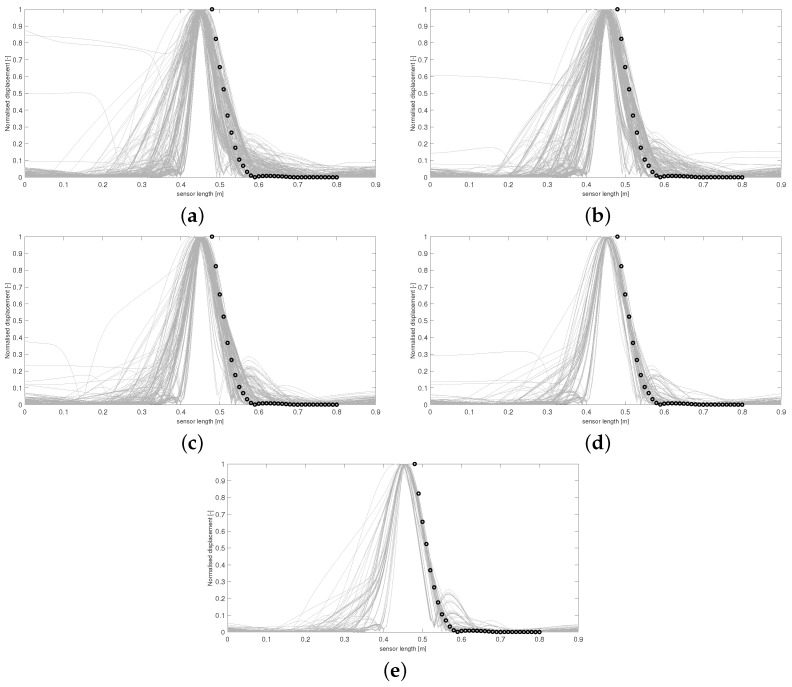
Accepted curves for each wave of ABC-SMC using a difference of area metric. Curves in (**a**–**e**) correspond to accepted artificial data $Y^{*}$ from waves 1–5, respectively. Experimental data are shown with circle markers.

**Figure 4:**
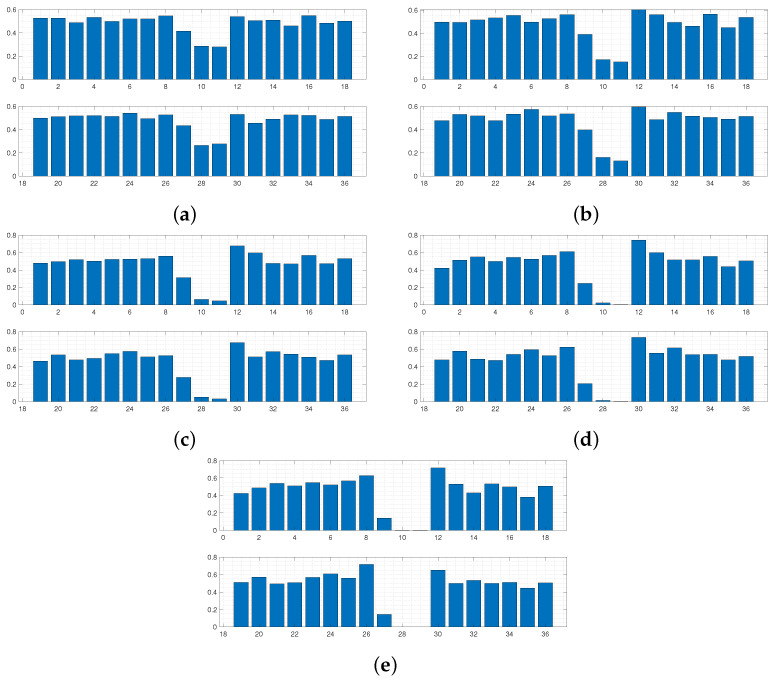
Intermediate distributions for each dimension of θ for each wave of ABC-SMC, using a difference of area metric. Distributions in (**a**–**e**) correspond to belief of fixity of each boundary condition after each wave 1–5, respectively.

**Figure 5:**
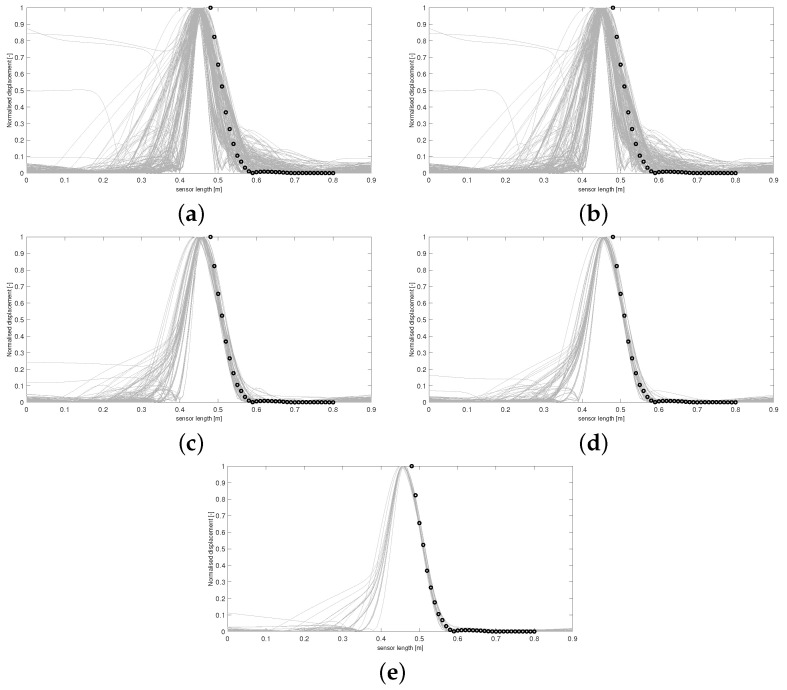
Accepted curves for each wave of ABC-SMC using a least squared metric. Curves in (**a**–**e**) correspond to accepted artificial data $Y^{*}$ from waves 1–5, respectively. Experimental data are shown with circle markers.

**Figure 6:**
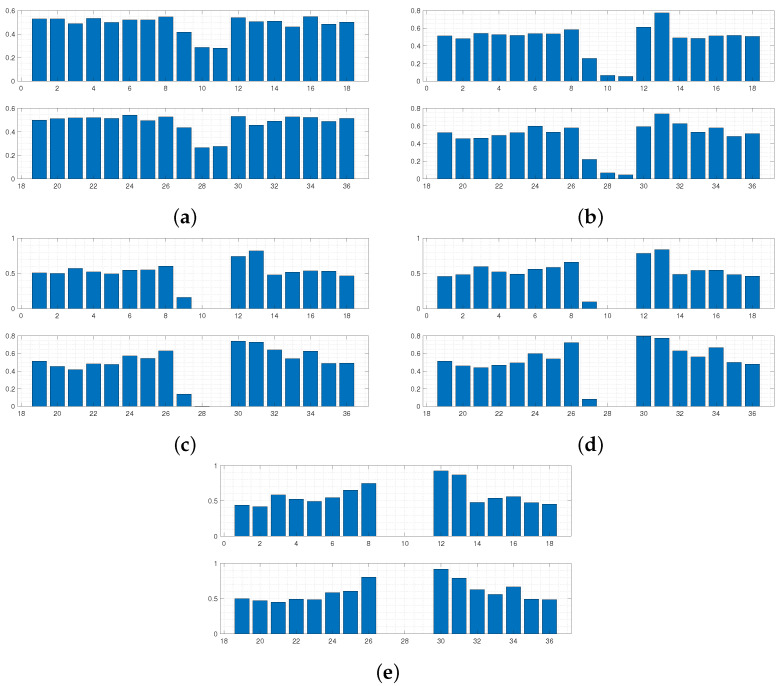
Intermediate distributions for each dimension of θ for each wave of ABC-SMC, using a least squared metric. Distributions in (**a**–**e**) correspond to belief of fixity of each boundary condition after each wave 1–5, respectively.

Using this form of *D* allows the areas of the respective curves to be compared, and if $D(Y,Y^{*})\leq \varepsilon$, the 36 boundary conditions producing $Y^{*}$ are accepted. Figure 3 and Figure 4 display results from this run. The results in Figure 5 and Figure 6 were produced by using a least squared metric on the same experimental data. The metric used in this instance takes the form

(5) $$D\left(Y,Y^{*}\right)=\sum_{i=0}^{33}|y_{48+i}^{*}-y_{48+i}{|}^{2}.$$

The terms $y_{j},y_{j}^{*}$ are the values of the experimental and simulated curves $Y,Y^{*}$ at *j* cm along the sensor length, respectively. Note, as data were only collected along a subsection of the sensor length, it is only over this interval that areas of simulated data and least squared distance are calculated for each run. The 500 normalised simulated data $Y^{*}$ accepted on each wave are displayed first in Figure 3. The corresponding probability of each of the 36 boundary conditions being fixed after all 500 samples were accepted for each wave is displayed in Figure 4.

It can be observed in Figure 3 that the sequence of accepted curves more closely approximates the shape of the experimental data for successive waves. The corresponding boundary conditions capable of producing these more acceptable data show a trend of favouring free conditions at the shaker impact area. By wave 5 (Figure 3e and Figure 4e), it appears that only configurations of conditions that are free at the shaker impact area are capable of producing curves within the margin of error that has been set for wave 5.

For any ABC scheme, the metric utilised needs to be carefully chosen to fully incorporate the notion of ‘similarity’ between the objects *Y* and $Y^{*}$ being considered. If the outputs of the system in question were real numbers, then a simple function such as the absolute distance $D\left(Y,Y^{*}\right)=\left|Y-Y^{*}\right|$ between the two data would be a perfect mechanism to classify similarity. In this scenario, D=0 would imply $Y=Y^{*}$, and so it is understood that the parameters θ producing $Y^{*}$ must also produce *Y*. Unfortunately, for most cases where ABC schemes are applicable, the parameters to be inferred do not produce objects as simple as this. Understanding the objects being compared is important, i.e., curves in $R^{2}$ in the current scenario. However, the metric needs to fully reflect what properties of the experimental data the user wishes to emulate. For the presented case, the user wishes to recover simulated absolute maximum displacement profiles of the plate that match, as closely as possible, the trajectory of the data in Figure 1. Whilst taking the form of *D* in Equation (4) provided the expected inference, it is clear from Figure 3e that boundary conditions are still being accepted that produce curves with secondary peaks, even though the target data do not display this feature. The difference between the areas of these curves and the experimental curve are still within the margin of error that has been set, and so the scheme sees the samples generating these curves as appropriate, even though these curves do not follow a trajectory similar to *Y*. Thus, *D* has, in a sense, failed to only accept samples producing simulated data that conform to the conditions being targeted within five waves and more iterations would be needed to produce artificial data more closely approximating experimental data.

It was believed that using the form of *D* in Equation (5) would provide more sound inference when attempting to recover the exact trajectory of *Y* without the need to increase the number of waves, as this metric aims to minimise the sum of offsets between the two sets of data being considered, ensuring that simulated curves with similar trajectories to *Y* produce lower values of *D*.

Utilising a least squared metric as seen in Figure 5 and Figure 6 accounted more for the shape of the simulated curves. It was believed that this form of *D* would penalise curves with secondary peaks more heavily when being compared against the experimental data, which consists of only a single thin peak. In later waves, this was hypothesised to help in refining the space of appropriate samples, leading to a more accurate approximate posterior distribution. It can be seen by wave 5 that no curves are accepted with secondary peaks along the points covering the experimental data curve. Intense overlapping of the accepted curves by the final wave in Figure 5 displays that for an appropriate choice of metric, more realistic samples can be recovered, ensuring a more accurate form of the final approximate posterior distribution.

Regardless of the slight improvement in results for the second run, both instances arrive at the conclusion that the clamps surrounding the midsection of the sensor must be loose. It can also be seen that clamps 8, 12, 13, 26, 30, and 31 have a much higher probability of being fixed. So it must be the case that these clamps were deemed fixed in a greater proportion of elements sampled from Θ producing accepted simulated curves, implying it is highly likely that these clamps are in fact fixing the corresponding boundary conditions in the experimental setup, constraining the width of this peak in Figure 1. As stated in the introduction, coupling between the clamping system and the sensor varies between sections, and it can be seen that combinatorial ABC-SMC has provided valuable insight into the true nature of this coupling.

For the final waves in Figure 3 and Figure 5, it can be seen that away from the impact area, the clamps’ probability of being fixed or free is confined within the [40,60]% interval, and so the algorithm provides less certain inference in these areas. This phenomenon occurs due to the fact that the displacement would diminish over a relatively short length scale (for any boundary condition configuration) along the sensor when considering the structure (three layers) and material properties of the sensor (as can be seen in Figure 1). Consequently, the clamping configuration in these further away areas would have minimal effect on the displacement profile.

**Figure 7:**
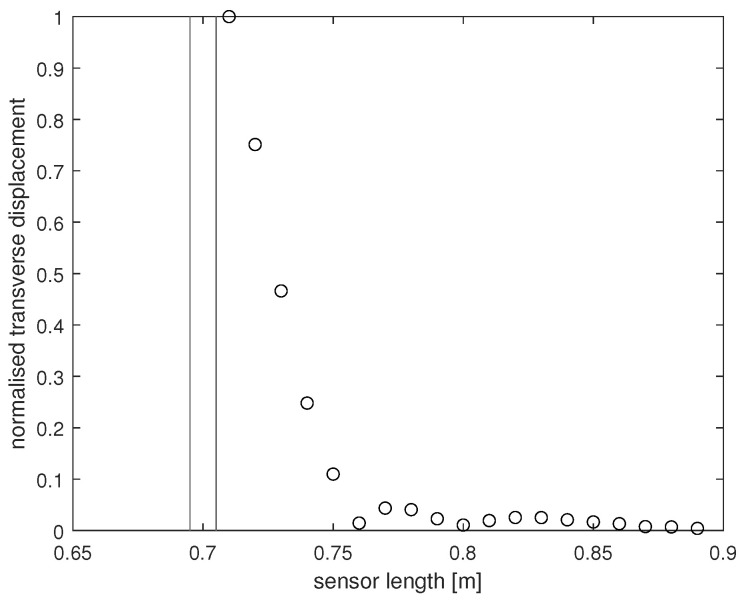
Normalised absolute displacement profile for the sensor subjected to mechanical excitation of 0.035–0.037 N operating at 5 Hz. Impact located at 70 cm along the 90 cm sensor length.

While these results convey the applicability of combinatorial ABC-SMC for providing inference of the coupling variation between the clamping system and the sensor, it was necessary to verify the model used for this work. Additional data were obtained from a variation of the original experimental setup at LVV. These data were collected from a similar experiment, where the impact was located at 70 cm along the length of the sensor, rather than 45 cm. Combinatorial ABC-SMC was applied to these data, displayed in Figure 7. It was the aim to provide insight on how the more complex looking data manifest in the approximate posterior at the end of wave 5 in this additional run.

One can see how the maximum displacement profiles presented in Figure 1 and Figure 7 are nothing alike. These data were chosen specifically to see if the performance of combinatorial ABC-SMC would yield a similar posterior distribution of boundary conditions for the data in Figure 1. Additionally, it was believed that the existence of secondary and third peaks in Figure 7 may be due to more variation in coupling between neighbouring clamps in this section of the sensor, as well as the fact that there is greater displacement of the sensor further from the shaker location in Figure 7 compared to Figure 1.

**Figure 8:**
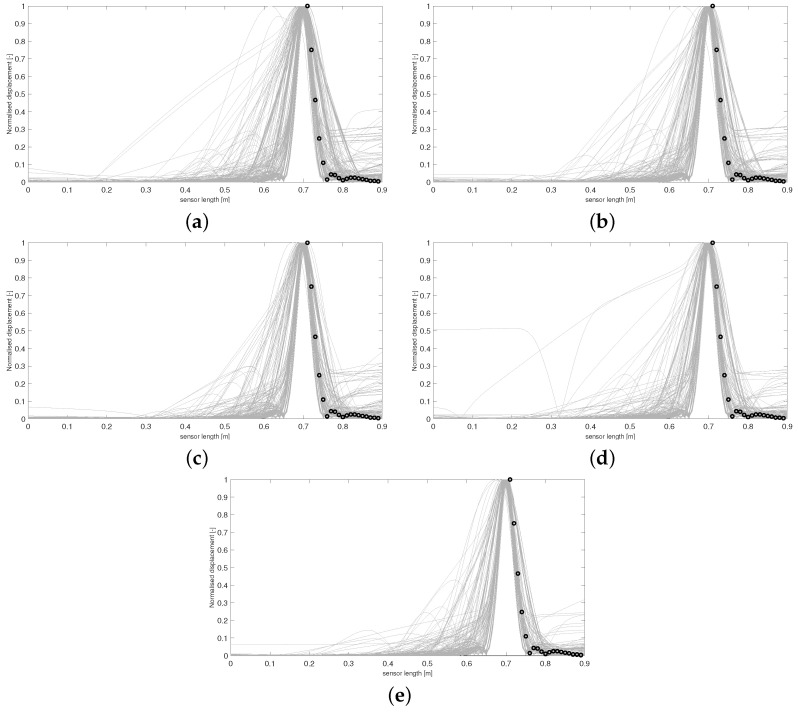
Accepted curves for each wave of ABC-SMC using a least squared metric applied to data from Figure 7. Curves in (**a**–**e**) correspond to accepted artificial data $Y^{*}$ from waves 1–5, respectively. Experimental data are shown with circle markers.

**Figure 9:**
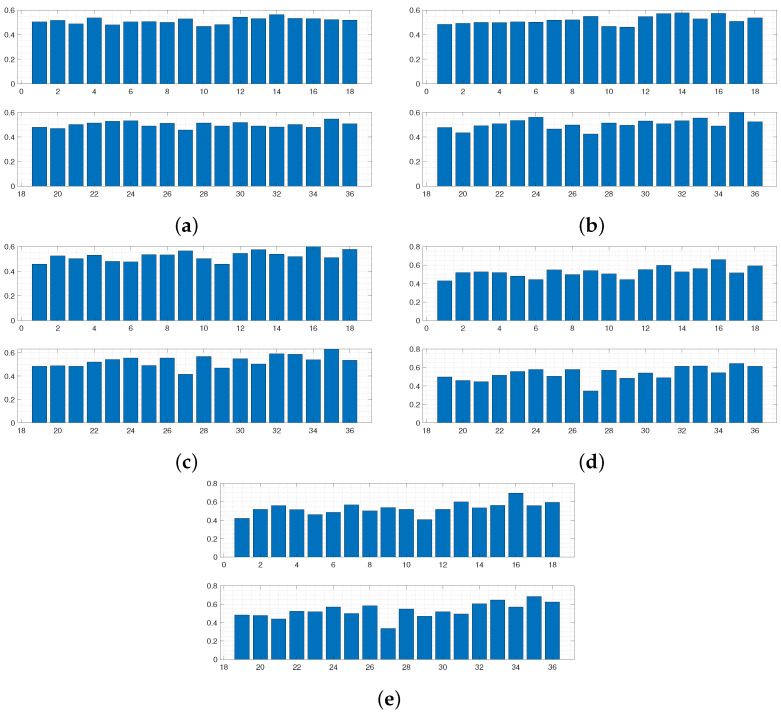
Intermediate distributions for each dimension of θ for each wave of ABC-SMC, using a least squared metric applied to data from Figure 7. Distributions in (**a**–**e**) correspond to belief of fixity of each boundary condition after each wave 1–5, respectively.

Data from Figure 7 were only collected in 1 cm intervals starting from 4 cm away from the shaker position, unlike previous data, which started 3 cm from the shaker position. The MATLAB optimisation toolbox was used to fit the data at 74–76 cm to a Gaussian function centred at the shaker impact location. This was performed using the lsqcurvefit function to replicate the shape of the displacement curve localised to the impact area. Using the combined LDV data and artifical data, a longer displacement profile on which to apply combinatorial ABC-SMC was produced. Equipped with a metric of the form shown in Equation (5), combinatorial ABC-SMC was applied to the data in Figure 7. This was implemented to provide a comparison of how well the aforementioned metric would perform on two different datasets. Due to the increased complexity of experimental data in this case, it was hypothesised that there would be differing levels of convergence between the two runs. It was the authors’ belief that the recovery of samples closely approximating the data in Figure 7 may require additional metric structure to produce samples with the more complex trajectory seen in the data, which a standard least squared metric similar to that in Equation (5) would ignore. Results from this run are displayed in Figure 8 and Figure 9.

From the results in Figure 8 and Figure 9, it can be observed that the least squared metric was unable to provide any meaningful inference from five successive waves of sampling on the sensor boundary conditions in locations where experimental data were collected. As outlined previously, correct inference of the target parameters relies heavily on an appropriate metric structure capable of recovering the features of importance contained in the experimental data. A metric globally minimising the average of vertical offsets for the data in Figure 7 clearly does not account for the multiple peaks seen in the data, and so falls short in mimicking the experimental data. This is comparable to the failings of the metric in Equation (4). Additional structure is needed to fully capture the local variations seen in the secondary and third peaks, whereas a metric of the form in Equation (5) is only capable of minimising the global vertical offsets between two datasets.

To combat poor convergence, the metric used for acceptance was modified significantly to account for the additional structure seen in Figure 7. The authors hypothesised that combinatorial ABC-SMC would then detail what boundary conditions would be necessary to produce additional peaks in displacement as seen in Figure 7, provided the metric was tailored appropriately to the specific data. When considering how to assess samples, the input data were split into 3 sections: 71–75 cm, 76–80 cm, and 81–89 cm. Again, a least squared metric was used, but each of these sections was considered separately. This ensured the squared vertical distance between simulated and experimental data was averaged for subdomains of the plate length rather than the whole length itself. Additional terms were added to the metric, ensuring only curves displaying a gradient increase and decrease in the 76–80 cm region were accepted, to encourage more samples to be accepted displaying secondary peaks. The metric *D* used for this run takes the form

(6) $$D\left(Y,Y^{*}\right)=\left(\sum_{i=0}^{4}|y_{71+i}^{*}-y_{71+i}{|}^{2},\sum_{i=0}^{4}|y_{76+i}^{*}-y_{76+i}{|}^{2},\sum_{i=0}^{8}|y_{81+i}^{*}-y_{81+i}{|}^{2},H_{\left\{1\right\}},H_{\left\{2\right\}}\right),$$

where

(7) $$H_{\left\{1\right\}}=\left\{\begin{array}{c} 1\;\mathrm{if}\;y_{78}^{*}-y_{76}^{*}>0,\\ 0\;\mathrm{if}\;y_{78}^{*}-y_{76}^{*}<0 \end{array}\right.,\;H_{\left\{2\right\}}=\left\{\begin{array}{c} 1\;\mathrm{if}\;y_{80}^{*}-y_{78}^{*}<0,\\ 0\;\mathrm{if}\;y_{80}^{*}-y_{78}^{*}>0 \end{array}\right..$$

**Figure 10:**
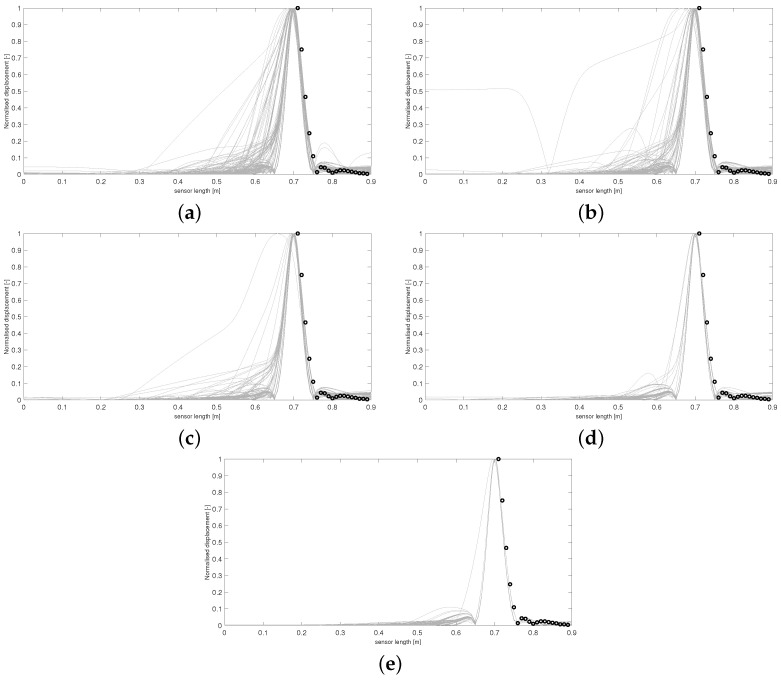
Accepted curves for each wave of ABC-SMC using a split least squared metric. Curves in (**a**–**e**) correspond to accepted artificial data $Y^{*}$ from waves 1–5, respectively. Experimental data are shown with circle markers.

**Figure 11:**
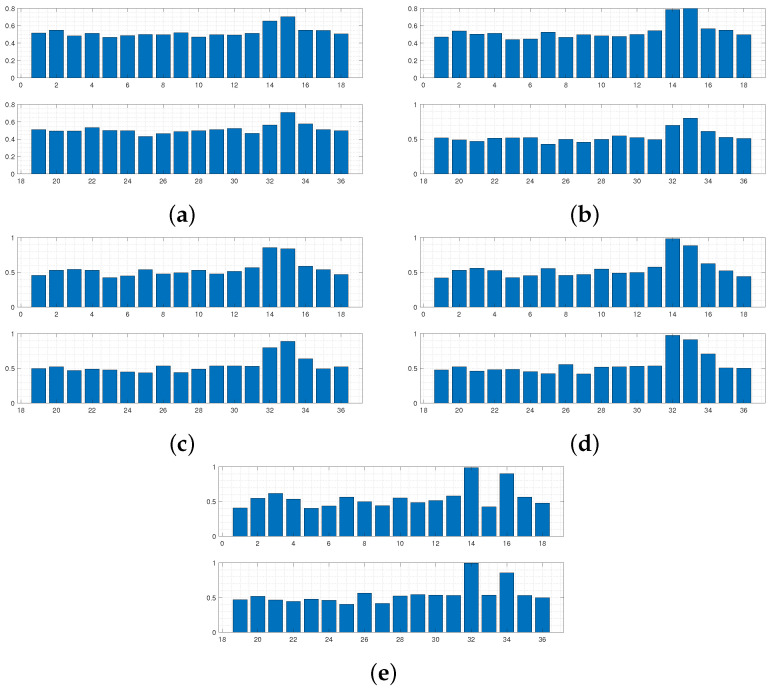
Intermediate distributions for each dimension of θ for each wave of ABC-SMC, using a split least squared metric. Distributions in (**a**–**e**) correspond to belief of fixity of each boundary condition after each wave 1–5, respectively.

The terms $y_{j},y_{j}^{*}$ are the values of the experimental and simulated curves $Y,Y^{*}$ at *j* cm along the sensor length, respectively. The terms in Equation (7) characterise whether there is a secondary peak in the 76-80cm region of $Y^{*}$. Both $H_{\left\{1\right\}},H_{\left\{2\right\}}$ must be equal to one for this condition to be satisfied. The results for this run are displayed in Figure 10 and Figure 11.

As with the previous converging runs, progressing through the waves, accepted simulated data mimic more closely the trajectory of the experimental data as desired, with a large reduction in variance in the final two waves. In the first four waves, results appear to show that clamps 14, 15, 32, and 33, which are positioned between 65 and 75 cm along the sensor length, have a high probability of being fixed. This is in contrast to all of the other clamps, which appear to have little effect on the simulated data, as all of these elements have an equal probability of being fixed or free, even by wave 4. However, the stricter margin of error present in wave 5 results in only much more realistic curves being accepted. This improvement can be seen by the lower variance in the values of the accepted curves in the 75–90 cm interval. Consequently, the posterior changes quite significantly compared to the previous waves as well. Clamps 14 and 32 at the 65–70 cm position are deemed completely fixed in this final wave, which continues the trend being shown throughout the previous waves. Clamps 15 and 33 at the 70–75 cm position appear to have little effect on the sensor response, despite the shaker position covering this area. Instead, clamps 16 and 34 are now deemed almost certainly fixed. This covers the positions of 75–80 cm along the sensor, which is the location of the secondary peak. The authors conclude that clamps 16 and 34 being fixed was most likely a limiting factor in the height of this secondary peak. Additionally, this also played an important role in constraining the width of the main peak.

Due to limitations and time constraints in the experimental work, LDV data were not collected over the entire length of the sensor. The algorithm has freedom in how to choose the clamps conditions in the ‘blind’ areas where experimental data are not recorded, as the algorithm would not have anything to compare the simulated displacement to at these points. This may be a cause for concern when evaluating the accuracy of the formed posterior distribution in each run, as the sensor response in these unrecorded areas may provide a whole picture of the boundary condition configuration for the entire sensor. The counterpoint to this assertion is that it seems reasonable to have very little change from the prior belief in regions far from the measurements. However, in all cases, the absolute displacement of the sensor diminishes over a very short length as can be seen in Figure 1 and Figure 7, making it difficult to infer the state of clamping in areas further away from the impact location.

## 6. Conclusions

Through the several sets of presented results, it has been shown that combinatorial ABC-SMC can be successfully applied to resolve uncertainty in calibrating the challenging boundary conditions of this fibre-optic sensor. The procedure is also generally applicable to cases where a calibration of a set of binary variables is needed. The algorithm determines the most realistic configurations of clamping of the fibre-optic sensor given the observed displacement profile when subjected to localised mechanical excitation. The obtained results match reasonable expectations of how the sensor would respond dynamically in different scenarios, subsequently providing inference on the underlying boundary condition parameter θ responsible for the sensor dynamic response. Future work could include the repetition of experiments with another impact location, or recording LDV data along the entire sensor length to observe if additional information could lead to a tighter posterior distribution. The formed posteriors at the end of wave 5 for each run result in roughly symmetrical boundary conditions along each side of the sensor. The authors believe that the size of the parameter space may be considerably reduced if only symmetrical boundary conditions are considered, further reducing the computational cost required for additional runs, even if additional experimental data are collected along the entire plate length.

In its current form, combinatorial ABC-SMC assumes independence between the elements of θ. However, since the elements of θ are likely correlated, some refinement of the proposal could increase the efficiency of the sampler further. Despite the assumptions seen in this modelling approach, it has been seen how in a challenging inference setting, the presented approach has allowed insight into a complex modelling procedure, highlighting areas both of confidence and uncertainty.

## Data Availability

The raw data supporting the conclusions of this article will be made available by the authors on request.
